# Deep learning model to predict COPD hospital admissions based on meteorological data: a medical meteorological forecast

**DOI:** 10.3389/fdata.2026.1687969

**Published:** 2026-06-18

**Authors:** Lei Zhang, Mingjie Zhang, Jinghong Zhang, Yajie Zhang, Tian Xie, Yipeng Ding, Shuyuan Chu, Haihong Wu

**Affiliations:** 1 Department of Pulmonary and Critical Care Medicine, Hainan Affiliated Hospital of Hainan Medical University, Hainan General Hospital, Haikou, Hainan, China; 2 Key Laboratory of South China Sea Meteorological Disaster Prevention and Mitigation of Hainan Province, Climate Center of Hainan Province, Haikou, Hainan, China; 3 Department of General Practice, Hainan General Hospital, Hainan Affiliated Hospital of Hainan Medical University, Haikou, Hainan, China; 4 Guangxi Clinical Research Center for Diabetes and Metabolic Diseases, The Second Affiliated Hospital of Guilin Medical University, Guilin, Guangxi, China; 5 Guangxi Key Laboratory of Metabolic Reprogramming and Intelligent Medical Engineering for Chronic Diseases, The Second Affiliated Hospital of Guilin Medical University, Guilin, Guangxi, China; 6 Department of Pulmonary and Critical Care Medicine, The Second Affiliated Hospital of Guilin Medical University, Guilin, Guangxi, China

**Keywords:** chronic obstructive pulmonary disease (COPD), deep leaning, Long Short-Term Memory (LSTM), medical meteorological forecast, meteorological condition

## Abstract

**Background:**

Chronic obstructive pulmonary disease (COPD) has placed a substantial health burden on the world. Meteorological conditions are associated with hospital admissions for COPD. In this study, we aim to develop a model of medical meteorological forecasting for COPD hospital admissions.

**Methods:**

A predictive model was developed using a Long Short-Term Memory (LSTM) algorithm applied to time series data on COPD hospital admissions and meteorological conditions. Data were collected daily from 25 September 2016 to 26 December 2020. Performance of the model was assessed using the mean squared error (MSE), root mean squared error (RMSE), mean absolute error (MAE), and *R*^2^. The association between the risk of COPD hospital admissions and meteorological conditions was assessed using a conditional logistic regression analysis and a conditional Poisson regression analysis in a time-stratified case-crossover design.

**Results:**

A total of 17,555 hospital admissions for COPD from 1 January 2017 to 31 December 2019 were included in the final LSTM model. Regarding the performance of the LSTM model, the MSE was 0.028, the RMSE was 0.167, the MAE was 0.134, and *R*^2^ was 0.416. Regression analysis revealed that the maximum temperature was positively associated with COPD hospital admissions.

**Conclusion:**

The LSTM model offers potential for medical meteorological forecasting to predict COPD hospital admissions among the general population according to the local climate. Higher maximum temperature may be a risk factor for COPD hospital admissions.

## 1 Instruction

Chronic obstructive pulmonary disease (COPD) has placed a substantial health burden on the world. The global prevalence of COPD among people aged 30–79 years was 10.3%, translating to 391.9 million people in 2019 (Adeloye et al., 2022). COPD is the fourth leading cause of death worldwide, causing 3.5 million deaths in 2021, which accounts for approximately 5% of all global deaths (World Health Organization, 2025). In China, the estimated prevalence of COPD was 13.6% among individuals aged 40 years or older, affecting nearly 100 million people (Fang et al., 2018).

Meteorological conditions are associated with COPD hospital admissions (Romaszko-Wojtowicz et al., 2025). Thus, a health forecast for COPD hospital admissions based on meteorological conditions would benefit at-risk populations and health institutions from a public health perspective (Ghazvinian and Karami, 2024). In our study, we developed a predictive model to forecast hospital admissions for COPD based on meteorological conditions. Although low temperature is associated with an increased risk of acute exacerbation of chronic obstructive pulmonary disease (AECOPD) hospital admissions (Yang et al., 2024), the average number of COPD hospital admissions was significantly increased in summer (Romaszko-Wojtowicz et al., 2025). Therefore, we developed a predictive model for COPD admissions based on meteorological conditions in a district of tropical monsoon climate, Haikou, Southern China. The tropical monsoon climate, also called the tropical wet climate or trade-wind littoral climate, presents warm temperatures throughout the year, low pressure, and abundant rainfall concentrated in the high-sun season (Thamminidi, 2022). It provides an ideal setting to explore the association between COPD hospital admissions and a warm climate.

In this study, the predictive model was developed using the Long Short-Term Memory (LSTM) algorithm. LSTM is a recurrent network architecture combined with an appropriate gradient-based learning algorithm (Houdt et al., 2020). It can capture long-term dependencies in sequential data, which is ideal for time series forecasting (Houdt et al., 2020). LSTM has been used to develop medical meteorological forecasts and has shown good performance (Yang et al., 2025). To compare it with the classical machine learning algorithm, we also developed a predictive model using the random forest (RF) regression algorithm. Moreover, we investigated the association between COPD hospital admissions and meteorological characteristics in a time-stratified case-crossover design (Wu et al., 2021; Janes et al., 2005; Rai et al., 2023). The time-stratified case-crossover design has been widely used for location-specific time series data (Kono et al., 2025). It is characterized by each individual serving as her or his own control (Wu et al., 2021).

Therefore, in this study, we developed predictive models as medical meteorological forecasts for COPD hospital admissions and further explored the association between COPD hospital admissions and meteorological conditions in a time-stratified case-crossover study.

## 2 Methods

### 2.1 Study population

This study collected data on COPD hospital admissions daily from 25 September 2016 to 26 December 2020 in Haikou. COPD was diagnosed according to J44 (chronic obstructive pulmonary disease) in the International Classification of Diseases, 10th Revision (ICD-10) classification. The data were extracted from medical records of comprehensive hospitals in Haikou, including Hainan General Hospital, the First Affiliated Hospital of Hainan Medical University, the Second Affiliated Hospital of Hainan Medical University, Hainan Traditional Chinese Medicine Hospital, Haikou Hospital of Traditional Chinese Medicine, the Third People's Hospital of Haikou, and the Fourth People's Hospital of Haikou. The study protocol was approved by the Medical Ethics Committee of Hainan General Hospital and adhered to the Declaration of Helsinki.

### 2.2 Meteorological data

The meteorological data for each day were collected from 25 September 2016 to 26 December 2020 in Haikou, consistent with the data for COPD hospital admissions. The variables included average atmospheric pressure, maximum atmospheric pressure, minimum atmospheric pressure, average temperature, maximum temperature, minimum temperature, average relative humidity, minimum relative humidity, precipitation, average wind velocity, and sunshine hours.

### 2.3 Modeling

**Figure 1:**
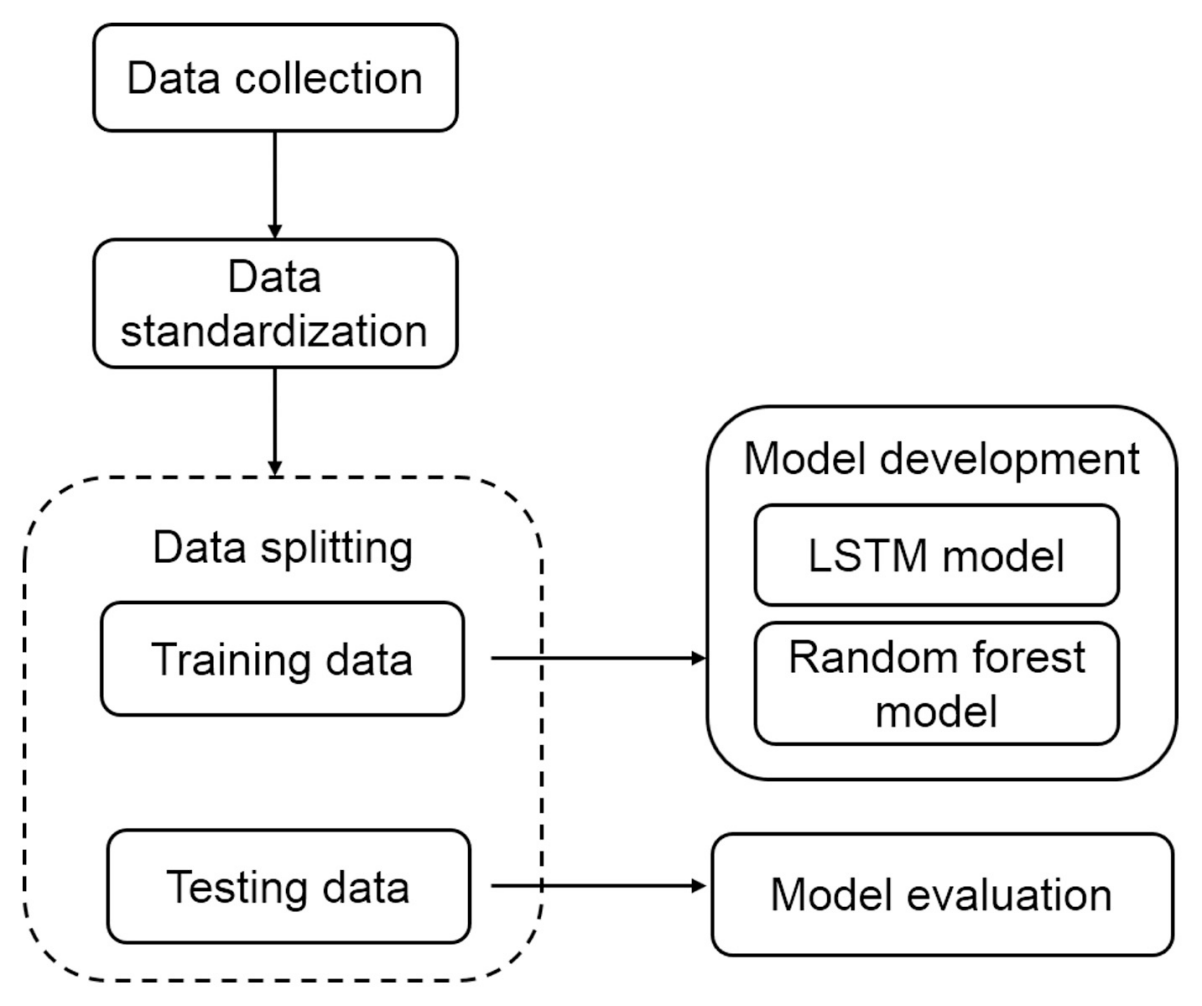
Flowchart of model construction. LSTM, Long Short-Term Memory.

The predictive model for COPD admissions on a given day was based on meteorological data. Data were standardized through minimum-maximum transformation. The first 80% of data were used as the training set to develop the LSTM and RF models, respectively. The last 20% of data were used as the testing set to evaluate the performance of the model. The hyperparameters for the training set were estimated using time series cross-validation with TimeSeriesSplit (n_split = 3) in the scikit-learn 22.1 library (https://scikit-learn.org/stable/) (Figure 1).

**Figure 2:**
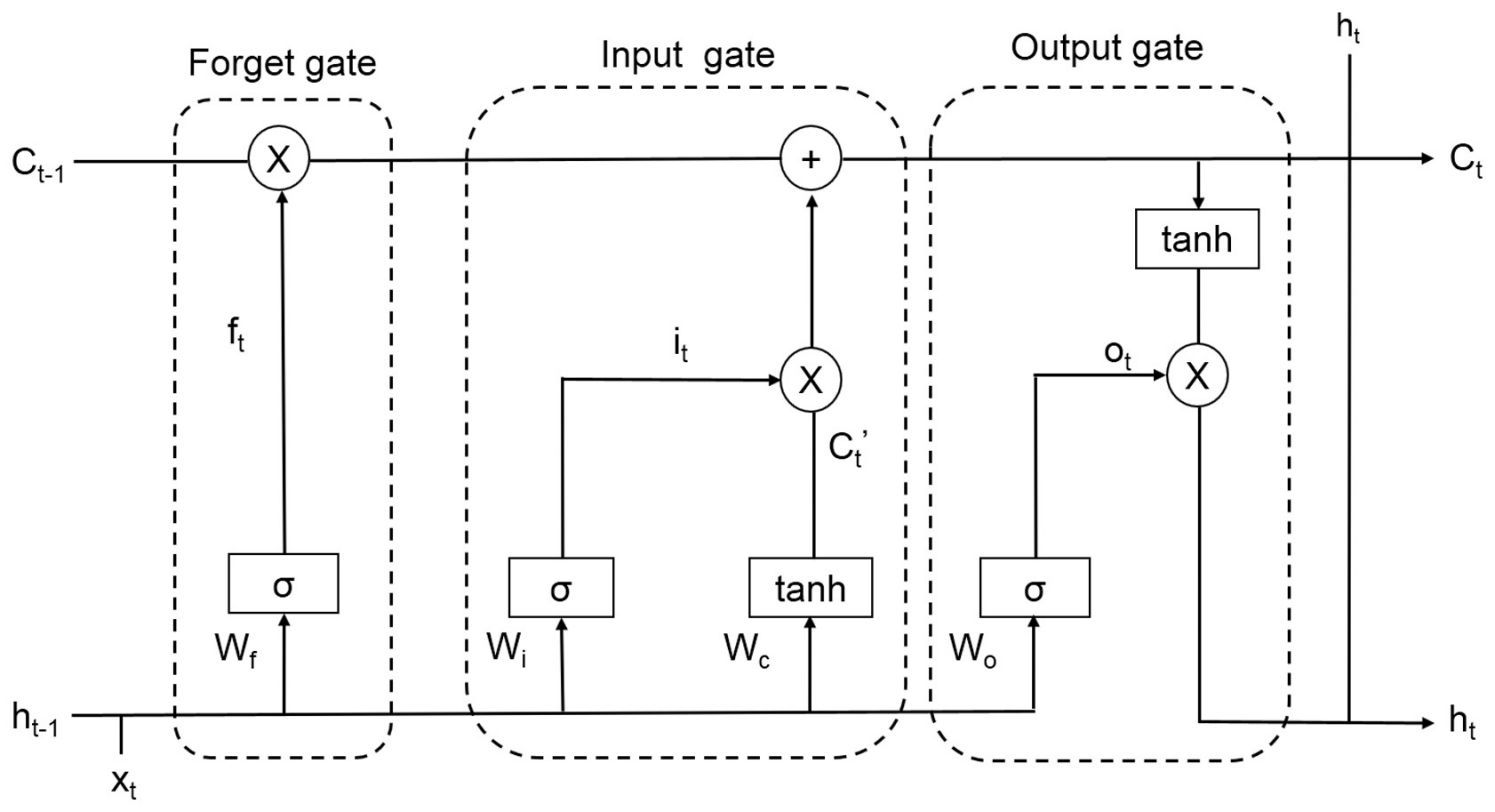
Long Short-Term Memory (LSTM) basic unit structure diagram.

The predictive model was developed using an LSTM algorithm for multi-step prediction (Figure 2). The gradient descent method was Adam. The Sigmoid function was the activation function for all LSTM layers and the output layer. The loss function was mean squared error (MSE). The candidate hyperparameters were as follows: LSTM units of 32 or 64, dense units of 64 or 128, dropout rate of 0.3, 0.4, or 0.5, and learning rate of 0.001 or 0.0001. The best parameter combination for the model was selected based on the lowest mean squared error (MSE). The model was developed in a Keras 2.3.1 environment using TensorFlow 2.0.1 as a backend and Python 3.7.6 (http://www.python.org). The MSE, root mean squared error (RMSE), mean absolute error (MAE), and *R*^2^ were used to assess the performance of the predictive model (Ghazvinian and Karami, 2025; Chen et al., 2025).

To compare the performance of a classical algorithm, a model using the RF regression algorithm was developed with Python 3.7.6 and the scikit-learn 22.1 library (https://scikit-learn.org/stable/). Grid search was used to estimate hyperparameters on the training set. The best parameter combination was selected based on the lowest MSE of the model. The r2_score was used to estimate the generalization score in tuning. The best value for n_estimators was first tuned using grid search, followed by max_depth. The min_samples_split and min_sample_leaf were tuned in combination. Finally, the max_features were tuned.

### 2.4 Study design and statistical analysis

The association between COPD hospital admissions and meteorological conditions was analyzed based on a time-stratified case-crossover study design (Wu et al., 2021; Janes et al., 2005; Rai et al., 2023). The date of hospital admission for COPD served as the case day. The days were selected from a monthly stratum window, namely 7, 14, and 21 days before and/or after the case day, resulting in 2–4 controls per case. In this study design, each case served as its own control and was adjusted for non-time and individual-level confounders, such as age, sex, smoking, and sociodemographic factors.

Both conditional logistic regression and conditional Poisson regression analyses were used to evaluate the association between COPD hospital admissions and meteorological conditions using the “season” and “gnm” packages in R 4.4.3. The results were presented as odds ratios (OR) and 95% confidence intervals (CI).

Chi-square test was used to assess the differences of patients' sex according to seasons. The differences of admission numbers and meteorological characteristics in four seasons were evaluated by one-way analysis of variance (ANOVA) followed by the Student—Newman—Keuls test or the Games—Howell test *P*-values of < 0.05 were considered to be statistically significant. All statistical analyses were conducted using SAS 9.4 (SAS Institute Inc., Cary, NC, USA).

## 3 Results

A total of 21,619 hospital admissions for COPD in a time series from 25 September 2016 to 26 December 2020 were collected in the study. Although there was a statistically significant difference in daily COPD hospital admissions among the four seasons (spring 14.1 ± 7.1, summer 13.4 ± 6.2, autumn 12.8 ± 7.9, and winter 15.5 ± 9.1), the difference was too small to be considered clinically meaningful in a metropolis with a population of three million. Male patients accounted for more than 80% of COPD hospital admissions. The mean age of patients at admission was approximately 75 years old (Table 1).

**Table 1:** COPD hospital admissions in seasons from 25 September 2016 to 26 December 2020.

Variables	Spring	Summer	Autumn	Winter	*P*-value
Inpatient number for COPD hospital admissions	14.1 ± 7.1^a^	13.4 ± 6.2^a^	12.8 ± 7.9^a^	15.5 ± 9.1	<0.001
Male (%)	4,191 (82.0)	3,900 (82.3)	4,381 (82.7)	4,549 (81.0)	0.112
Age (year)	75.3 ± 9.9	75.0 ± 10.2	75.4 ± 9.9	76.0 ± 9.6	<0.001

COPD, chronic obstructive pulmonary disease.

^a^Significantly different from winter.

**Figure 3:**
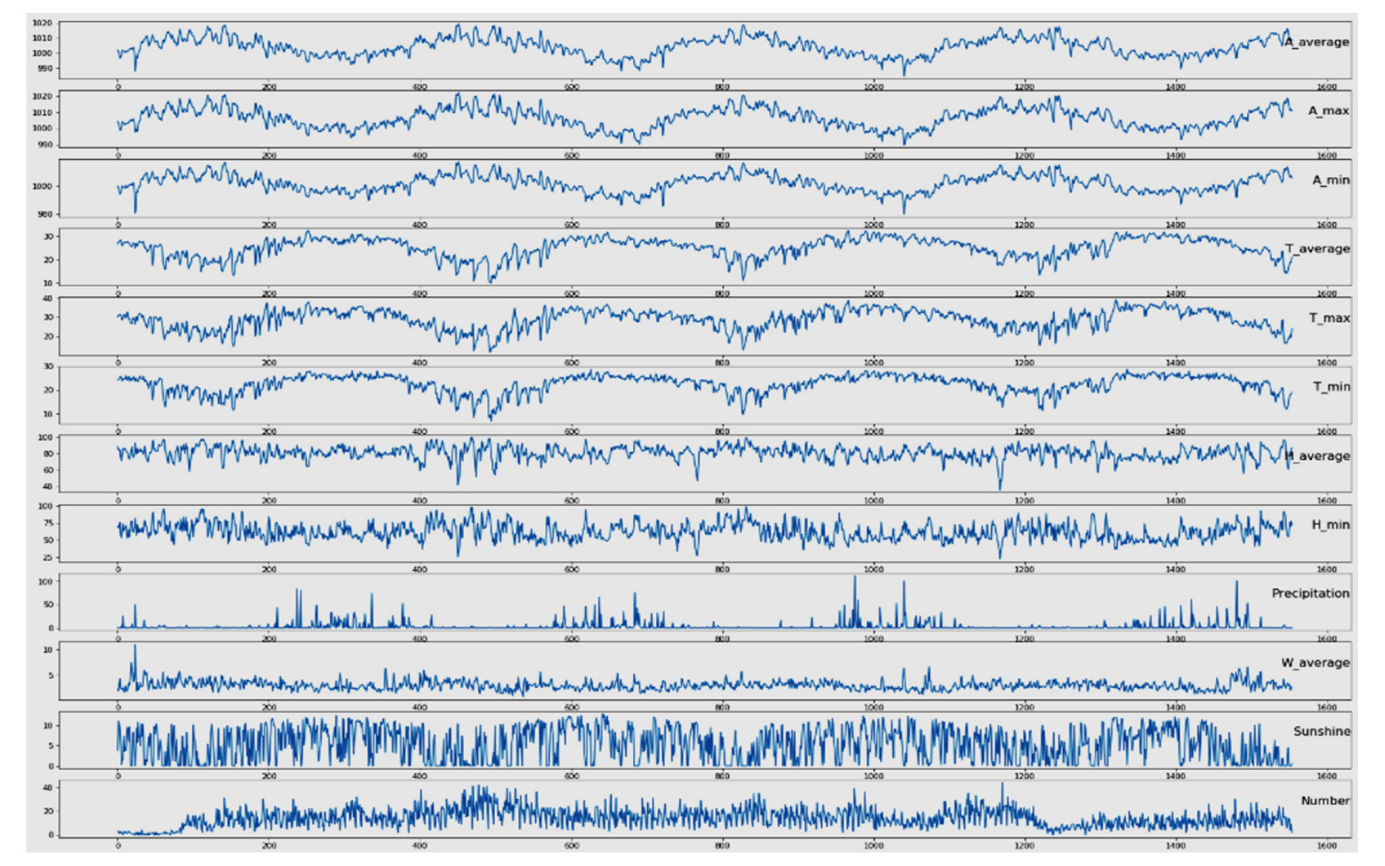
The pattern of meteorological conditions and COPD hospital admissions among the general population from 25 September 2016 to 26 December 2020 (a total of 1,554 days) in Haikou. The average atmospheric pressure, maximum atmospheric pressure, minimum atmospheric pressure, average temperature, maximum temperature, minimum temperature, and precipitation showed significant regularity as seasons. The number of COPD hospital admissions showed an increase in winter. A_average, average atmospheric pressure; A_max, maximum atmospheric pressure; A_min, minimum atmospheric pressure; T_average, average temperature; T_max, maximum temperature; T_min, minimum temperature; H_mean, average relative humidity; H_min, minimum relative humidity; W_average, average wind velocity; Sunshine, sunshine hours; number: inpatient number for COPD hospital admissions; COPD, chronic obstructive pulmonary disease.

As shown in Table 2, for the meteorological characteristics, the lowest levels of average atmospheric pressure, maximum atmospheric pressure, minimum atmospheric pressure, and average wind velocity were observed in summer, whereas the highest levels were recorded in winter. Moreover, our results showed the highest levels of average temperature, maximum temperature, minimum temperature, precipitation, and sunshine hours in summer but the lowest levels in winter. Both average and minimum relative humidity were highest in winter, followed by autumn, summer, and spring. The seasonal patterns of meteorological characteristics and COPD hospital admissions are shown in Figure 3.

**Table 2:** Climatological characteristics in seasons from 25 September 2016 to 26 December 2020.

Variables	Spring	Summer	Autumn	Winter	*P*-value
Average atmospheric pressure (hpa)	1003.9 ± 3.7^abc^	997.0 ± 2.8^bc^	1004.9 ± 4.3^c^	1010.8 ± 3.5	<0.001
Maximum atmospheric pressure (hpa)	1006.1 ± 3.9^abc^	998.7 ± 2.7^bc^	1006.9 ± 4.2^c^	1013.0 ± 3.6	<0.001
Minimum atmospheric pressure (hpa)	1001.4 ± 3.8^abc^	994.9 ± 2.9^bc^	1002.9 ± 4.6^c^	1008.6 ± 3.6	<0.001
Average temperature (°C)	25.9 ± 3.1^ac^	29.0 ± 1.5^bc^	25.7 ± 2.6^c^	19.8 ± 3.1	<0.001
Maximum temperature (°C)	30.4 ± 4.2^abc^	33.4 ± 2.3^bc^	28.8 ± 3.4^c^	22.7 ± 4.0	<0.001
Minimum temperature (°C)	23.0 ± 2.8^abc^	26.0 ± 1.1^bc^	23.4 ± 2.4^c^	17.9 ± 3.2	<0.001
Average relative humidity (%)	79.6 ± 6.8^c^	79.9 ± 6.5^c^	80.3 ± 7.5^c^	82.3 ± 10.1	<0.001
Minimum relative humidity (%)	58.6 ± 11.3^bc^	58.9 ± 9.7^bc^	63.6 ± 11.5^c^	68.3 ± 13.4	<0.001
Precipitation (mm)	2.7 ± 10.3^ac^	6.7 ± 13.4^bc^	3.2 ± 9.3^c^	0.4 ± 1.3	<0.001
Average wind velocity (m/s)	3.0 ± 0.7^ac^	2.6 ± 0.7^bc^	3.0 ± 1.1	3.1 ± 0.8	<0.001
Sunshine hours (h)	6.3 ± 3.9^bc^	6.8 ± 3.6^bc^	5.0 ± 3.9^c^	3.2 ± 3.4	<0.001

^a^Significantly different from summer.

^b^Significantly different from autumn.

^c^Significantly different from winter.

**Figure 4:**
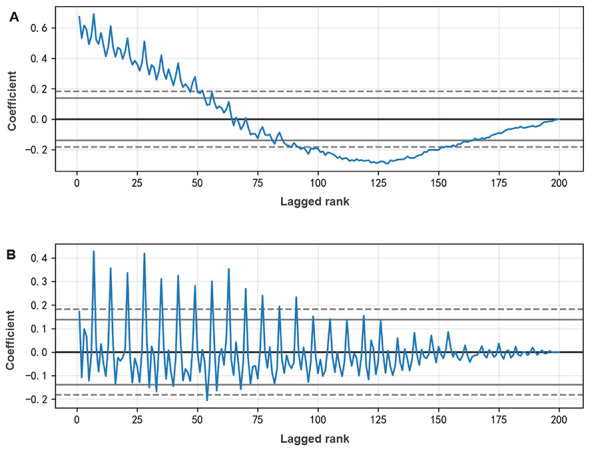
Lag effect in the models with the LSTM algorithm. **(A)** Model based on the data from 25 September 2016 to 26 December 2020. **(B)** Model based on the data from 1 January 2017 to 31 December 2019. LSTM: Long Short-Term Memory.

**Figure 5:**
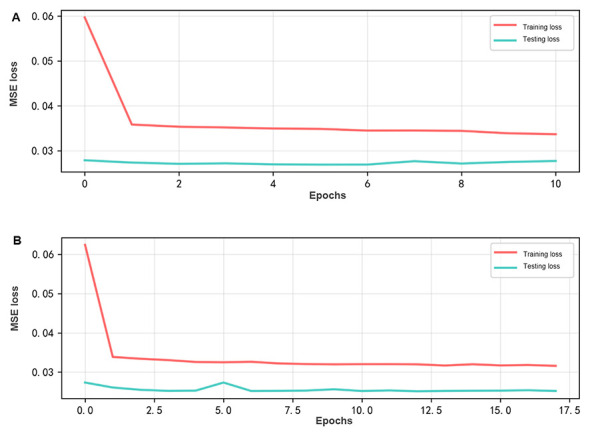
Loss value graphs in the models with the LSTM algorithm. **(A)** Model based on the data from 25 September 2016 to 26 December 2020. **(B)** Model based on the data from 1 January 2017 to 31 December 2019. LSTM: Long Short-Term Memory. MSE: mean squared error.

**Figure 6:**
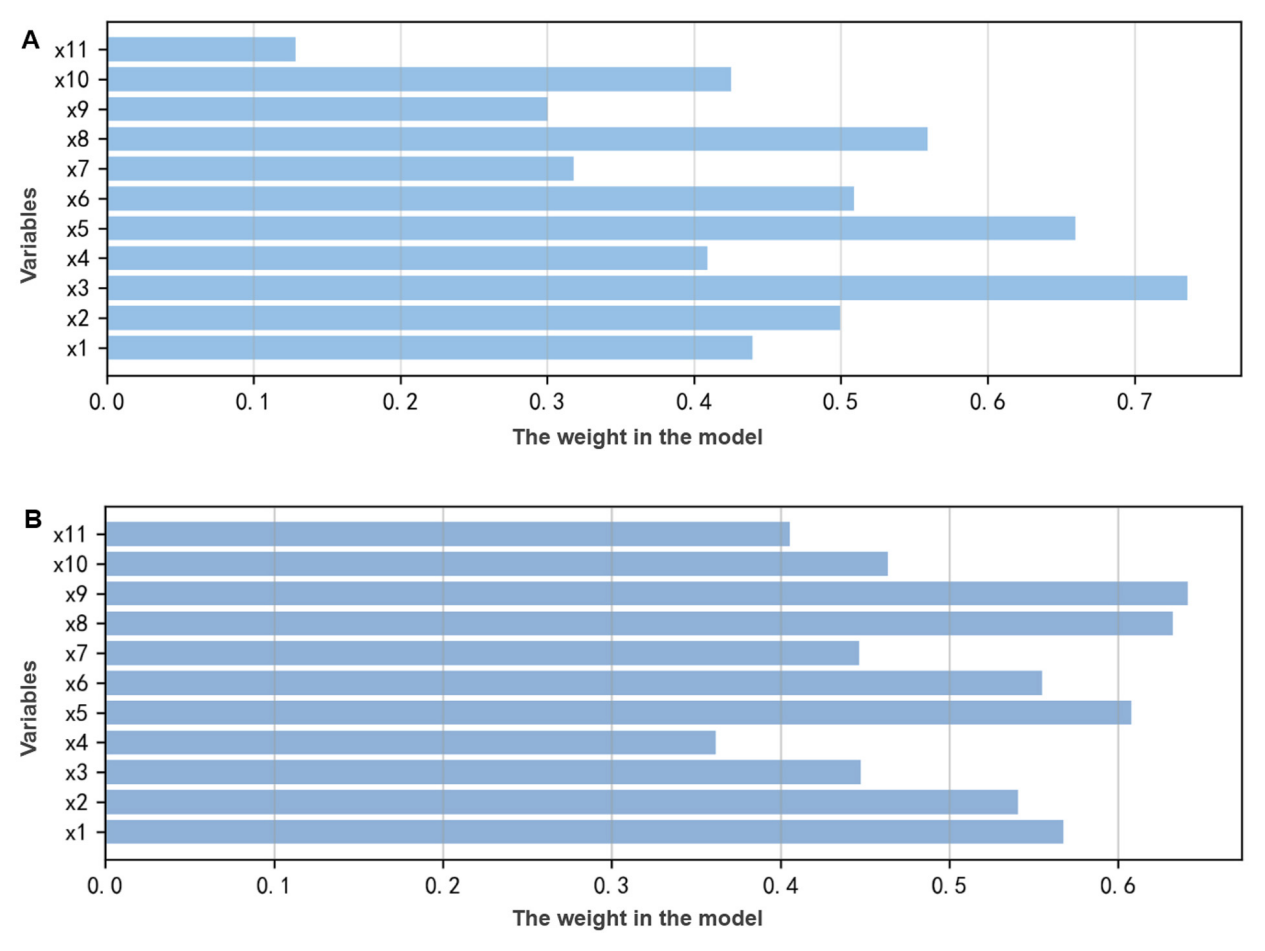
The importance of meteorological variables in the LSTM model. The weights in the figure were the sum of the absolute values in the output layer. **(A)** Model based on the data from 25 September 2016 to 26 December 2020. **(B)** Model based on the data from 1 January 2017 to 31 December 2019. LSTM, Long Short-Term Memory; MSE, mean squared error; x1, average atmospheric pressure; x2, maximum atmospheric pressure; x3, minimum atmospheric pressure; x4, average temperature; x5, maximum temperature; x6, minimum temperature; x7, average relative humidity; x8, minimum relative humidity; x9, precipitation; x10, average wind velocity; x11, sunshine hours.

The first predictive model was constructed using the LSTM algorithm, which included 21,619 COPD admissions from 25 September 2016 to 26 December 2020. In the model, as a result of hyperparameter selection, the time step was set to 3, prediction steps to 2, the first LSTM layer was 32, the second layer was 16, epochs were 20, and the batch size was 16. The performance of the model was as follows: The MSE was 0.022, and the RMSE was 0.148, indicating that the error in predicting the daily number of admissions was 0.148 using the LSTM model. The MAE was 0.124, and *R*^2^ was 0.017. The lag effect and loss value are, respectively, shown in Figures 4A, 5A. The importance of meteorological variables is shown in Figure 6A. The top five variables were minimum atmospheric pressure, maximum temperature, minimum relative humidity, minimum temperature, and maximum atmospheric pressure.

Since the first model included a special period of the coronavirus disease-2019 (COVID-19) pandemic from 2020, the performance, robustness, and extensive implementation of the model may be limited. Therefore, we developed the second model, which excluded data from 1 January 2020. The second model was based on the LSTM algorithm and included 17,555 COPD admissions from 1 January 2017 to 31 December 2019, covering a 3-year period. For the second model, the best hyperparameters were as follows: the time step was 3, prediction steps were 2, the first LSTM layer had 32 units, the second layer had 16, epochs were 20, and the batch size was 16. For the performance of the model, the MSE was 0.028, the RMSE was 0.167, the MAE was 0.134, and *R*^2^ was 0.416. The lag effect and loss value are, respectively, shown in Figures 4B, 5B. As illustrated in Figure 6B, the top five variables were precipitation, minimum relative humidity, maximum temperature, average atmospheric pressure, and minimum temperature.

A sensitivity analysis was performed for the second model. It excluded maximum temperature, which was borderline significant. For this model, the time step was 3, prediction steps were 2, the first LSTM layer had 32 units, the second layer had 16 units, epochs were 20, and the batch size was 16. The MSE was 0.028, the RMSE was 0.167, the MAE was 0.133, and *R*^2^ was 0.410. The performance of this model was similar to that of the model including maximum temperature.

The third model was developed using RF regression algorithm and included 17,555 COPD admissions from 1 January 2017 to 31 December 2019. Grid search revealed that the hyperparameters were n_estimators = 200, criterion = squared_error, max_depth = none, min_samples_split = 10, min_sample_leaf = 1, and max_features = 1. The MSE was 65.12 and the RMSE was 8.07. The performance of the RF model was considerably worse than that of the LSTM model.

The association between COPD hospital admissions and meteorological characteristics was assessed based on the conditional logistic regression analysis and the conditional Poisson regression analysis, respectively. With the data from 25 September 2016 to 26 December 2020, the maximum temperature was positively associated with COPD hospital admissions (conditional logistic regression: OR = 1.024, 95%CI = 1.001–1.048; conditional Poisson regression: OR = 1.024, 95%CI = 1.015–1.034) (Table 3). When excluding the period of COVID-19 and using data from 1 January 2017 to 31 December 2019 (Table 4), the maximum temperature was also positively associated with COPD hospital admissions (conditional Poisson regression: OR = 1.030, 95%CI = 1.020–1.040).

**Table 3:** Association between COPD hospital admissions and climatological variables from 25 September 2016 to 26 December 2020.

Climatological variables	Conditional logistic regression analysis		Conditional Poisson regression analysis	
	OR	95%CI	OR	95%CI
Average atmospheric pressure (hpa)	1.035	0.979–1.093	1.035	1.025–1.044
Maximum atmospheric pressure (hpa)	0.980	0.944–1.017	0.980	0.971–0.989
Minimum atmospheric pressure (hpa)	0.991	0.964–1.019	0.991	0.981–1.000
Average temperature (°C)	0.990	0.950–1.032	0.990	0.981–1.000
Maximum temperature (°C)	1.024	1.001–1.048	1.024	1.015–1.034
Minimum temperature (°C)	0.996	0.972–1.021	0.996	0.987–1.005
Average relative humidity (%)	0.997	0.992–1.002	0.997	0.987–1.006
Minimum relative humidity (%)	1.003	0.999–1.007	1.003	0.993–1.012
Precipitation (mm)	0.998	0.996–0.999	0.998	0.988–1.007
Average wind velocity (m/s)	0.988	0.966–1.011	0.988	0.979–0.998
Sunshine hours (h)	1.000	0.992–1.007	1.000	0.990–1.009

COPD, chronic obstructive pulmonary disease; OR, odds ratio; CI, confidence intervals.

**Table 4:** Association between COPD hospital admissions and climatological variables from 1 January 2017 to 31 December 2019.

Climatological variables	Conditional logistic regression analysis		Conditional Poisson regression analysis	
	OR	95%CI	OR	95%CI
Average atmospheric pressure (hpa)	1.030	0.970–1.093	1.030	1.020–1.040
Maximum atmospheric pressure (hpa)	0.977	0.938–1.017	0.977	0.967–0.986
Minimum atmospheric pressure (hpa)	0.998	0.969–1.029	0.998	0.989–1.008
Average temperature (°C)	1.005	0.960–1.053	1.005	0.996–1.015
Maximum temperature (°C)	1.023	0.997–1.050	1.023	1.013–1.033
Minimum temperature (°C)	0.981	0.955–1.007	0.981	0.972–0.990
Average relative humidity (%)	0.997	0.991–1.002	0.997	0.987–1.006
Minimum relative humidity (%)	1.003	0.998–1.008	1.003	0.993–1.013
Precipitation (mm)	0.997	0.995–1.000	0.997	0.988–1.007
Average wind velocity (m/s)	0.985	0.960–1.012	0.985	0.976–0.995
Sunshine hours (h)	0.998	0.990–1.005	0.998	0.988–1.007

COPD, chronic obstructive pulmonary disease; OR, odds ratio; CI, confidence intervals.

## 4 Discussion

In this study, we developed a predictive model as a medical meteorological forecast for COPD hospital admissions using an LSTM algorithm based on meteorological data in a district with tropical monsoon climate. Furthermore, we investigated the association between COPD hospital admissions and meteorological conditions in a time-stratified case-crossover design and found a positive association between COPD hospital admission and maximum temperature.

We developed a predictive model for COPD hospital admissions based on meteorological conditions. When data from the special period of COVID-19 in 2020 were excluded, the model may be more suitable for broader implementation. This model could be used as a medical meteorological forecast. The RMSE was 0.167, which indicates that the error of the predicted number in the daily number of admissions was 0.167 using the LSTM model. The performance of the LSTM model is substantially better than that of the machine learning model, namely RF, as shown in our study. Considering that Haikou is a metropolis that is home to nearly three million people and has seven large and comprehensive hospitals, this level of model performance may be sufficient for implementation as a medical meteorological forecast. Therefore, the LSTM model could be developed as a medical meteorological forecast in a district with a population scale similar to Haikou. The medical meteorological forecast could be helpful for health institutions to prepare for potential admissions, as well as for people at risk to prevent COPD hospital admissions. Our results indicate the potential for developing a medical meteorological forecast for COPD hospital admissions in a district of tropical monsoon climate with a large-scale population. Since our model was exploratory, it is necessary to externally validate it in future studies and test its generalizability.

In both LSTM models, with or without data from the COVID-19 period, minimum temperature and maximum temperature were among the top of the most important meteorological factors. It is widely accepted that low temperature is associated with an increased risk of COPD hospital admissions (Yang et al., 2024; Shi Z. et al., 2024). Interestingly, our conditional Poisson regression analysis showed a negative association between minimum temperature and the risk of COPD hospital admissions based on pre-COVID-19 data. This association may be related to the tropical monsoon climate in Haikou, where the minimum temperature is approximately 20 °C. This level of minimum temperature is relatively warm, which may influence the pathophysiological responses of human respiratory epithelial cells and immune cells to temperature (Li et al., 2025; Ge et al., 2013).

Our study has also found that the maximum temperature was positively associated with COPD hospital admissions. The results from our study suggest that high temperature may increase the risk of COPD hospital admissions in a district with tropical monsoon climate. This finding is consistent with a previous report, in which high temperature was associated with increased risk of COPD mortality in Southern China (Shi C. et al., 2024). The findings from a systematic analysis of Global Burden of Disease (GBD) study also show that the increasing trend of COPD burden may be attributable to high temperature exposure in low-and middle- sociodemographic index regions (Zou et al., 2022). The physiological mechanism leading to heat-related AECOPD is not yet fully understood. A suggested mechanism is the alteration of fluid and electrolytic balance in COPD patients (Gasparrini et al., 2015). Thus, in the district of tropical monsoon climate, such as Haikou, maximum temperature may lead to an increased risk of COPD hospital admissions.

Precipitation was the most important meteorological factor in the second LSTM model when excluding data from the COVID-19 period. According to the pattern of COPD hospital admissions and meteorological factors in our study, the decrease in COPD hospital admissions was associated with increased precipitation. The effect of increased precipitation on COPD hospital admissions may occur via an indirect pathway, in which precipitation influences air quality (Guo et al., 2016). Precipitation could help reduce PM2.5 pollution (Zhan et al., 2025), which can promote the onset of COPD (Wen et al., 2025; Kim et al., 2025).

In recent years, previous studies have reported the association between COPD hospital admissions and meteorology (Romaszko-Wojtowicz et al., 2025; Yang et al., 2024; Kazemi et al., 2024; Wang et al., 2020; Mészáros et al., 2015). The majority of previous studies have focused on air pollution. In contrast, our study focused on the climate itself. Second, our study explored a predictive model in a metropolis with a tropical monsoon climate, which has not been reported before. Moreover, the LSTM algorithm was applied in our model to predict COPD hospital admissions, which has not been reported in previous studies or in medical meteorological forecasting.

We acknowledge potential limitations in our study. First, air pollution was excluded from our model. Since short-term air pollution exposure is a risk factor for AECOPD hospital admissions (Lian et al., 2025), a model including air pollution may achieve higher accuracy than the present one. Second, the predictive model was based solely on meteorological conditions, without incorporating individual clinical characteristics. The model may achieve greater accuracy when individual characteristics are included. However, our model without individual characteristics is more broadly applicable than personal prediction when used as a medical meteorological forecast for the general population. Moreover, the reproducibility of the LSTM model could be influenced by the stochastic nature of optimization and the random initialization of weights in a neural network. Although it has been widely accepted that the LSTM model outperformed traditional forecasting methods, i.e., autoregressive integrated moving average (ARIMA), in forecasting time series (Siami-Namini et al., 2018), our results revealed that the LSTM model also greatly outperformed a typical machine learning algorithm, namely RF.

## 5 Conclusion

The LSTM model could be effective in predicting COPD hospital admissions among the general population. Therefore, the LSTM model provides potential for medical meteorological forecasting of COPD hospital admissions based on local climate. This model should be validated in future studies. Moreover, an increased maximum temperature may represent a risk factor for COPD hospital admissions.

## Data Availability

The raw data supporting the conclusions of this article will be made available by the authors, without undue reservation.
